# The primacy of multiparametric MRI in men with suspected prostate cancer

**DOI:** 10.1007/s00330-019-06166-z

**Published:** 2019-06-06

**Authors:** Jonathan Richenberg, Vibeke Løgager, Valeria Panebianco, Olivier Rouviere, Geert Villeirs, Ivo G. Schoots

**Affiliations:** 1 Department of Imaging, Brighton & Sussex University Hospitals NHS Trust and Brighton and Sussex Medical School, Brighton, BN2 5BE UK; 2 Department of Radiology, Herlev University Hospital Copenhagen University, Herlev, Denmark; 3 Department of Radiological Sciences, Oncology and Pathology, Sapienza, University of Rome, Rome, Italy; 4 Hospices civils de Lyon, Department of Urinary and Vascular Radiology, hôpital Édouard-Herriot, 69437 Lyon, France; 5 Faculté de médecine Lyon Est, Université Lyon 1, 69003 Lyon, France; 6 Department of Radiology, Ghent University Hospital, Ghent, Belgium; 7 Department of Radiology & Nuclear Medicine, Erasmus MC - University Medical Center Rotterdam, Rotterdam, The Netherlands

**Keywords:** Prostate cancer, Magnetic resonance imaging, Biopsy, Risk assessment, Observer variation

## Abstract

**Background:**

Multiparametric MRI (mpMRI) became recognised in investigating those with suspected prostate cancer between 2010 and 2012; in the USA, the preventative task force moratorium on PSA screening was a strong catalyst. In a few short years, it has been adopted into daily urological and oncological practice. The pace of clinical uptake, born along by countless papers proclaiming high accuracy in detecting clinically significant prostate cancer, has sparked much debate about the timing of mpMRI within the traditional biopsy-driven clinical pathways. There are strongly held opposing views on using mpMRI as a triage test regarding the need for biopsy and/or guiding the biopsy pattern.

**Objective:**

To review the evidence base and present a position paper on the role of mpMRI in the diagnosis and management of prostate cancer.

**Methods:**

A subgroup of experts from the ESUR Prostate MRI Working Group conducted literature review and face to face and electronic exchanges to draw up a position statement.

**Results:**

This paper considers diagnostic strategies for clinically significant prostate cancer; current national and international guidance; the impact of pre-biopsy mpMRI in detection of clinically significant and clinically insignificant neoplasms; the impact of pre-biopsy mpMRI on biopsy strategies and targeting; the notion of mpMRI within a wider risk evaluation on a patient by patient basis; the problems that beset mpMRI including inter-observer variability.

**Conclusions:**

The paper concludes with a set of suggestions for using mpMRI to influence who to biopsy and who not to biopsy at diagnosis.

**Key Points:**

*• Adopt mpMRI as the first, and primary, investigation in the workup of men with suspected prostate cancer.*

*• PI-RADS assessment categories 1 and 2 have a high negative predictive value in excluding significant disease, and systematic biopsy may be postponed, especially in men with low-risk of disease following additional risk stratification.*

*• PI-RADS assessment category lesions 4 and 5 should be targeted; PI-RADS assessment category lesion 3 may be biopsied as a target, as part of systematic biopsies or may be observed depending on risk stratification.*

**Electronic supplementary material:**

The online version of this article (10.1007/s00330-019-06166-z) contains supplementary material, which is available to authorized users.

## Introduction

In 2012, the European Society of Urogenital Radiology (ESUR) prostate committee promoted the use of multiparametric MRI (mpMRI) in the routine management of men with suspected or confirmed prostate cancer [1]. That proposal has gained widespread acceptance. The debate has now moved to *when* mpMRI should be used.

Expressions of interest were sought from the 58 members of the ESUR Prostate MRI Working Group at the European Congress of Radiology (ECR) in March 2017 in contributing to a position statement on the use of mpMRI in prostate cancer diagnosis. Each of the 7 initial positive respondents was invited to contribute but based on the relative contributions, the final author list was revised to 5 ensuring due representation of the group’s European composition; a sixth contributor joined at ECR, March 2018. The final contributors were from the UK, France, The Netherlands, Denmark, Italy, and Belgium.

The approach was to review published evidence, supplemented by knowledge of completed cohort studies in the process of being published (having been accepted for publication). In this way, there was very little intra-author disagreement. On the specific topic of biopsy planning—the pros and cons of systematic versus targeted-driven approach—there was some variation in how strongly the argument for targeted biopsy over systematic biopsy could be worded. As the paper neared completion, the results from on-going studies became known to the author group, such that a consensus position was reached.

## Evaluating clinically significant prostate cancer

### Evaluating clinically significant prostate cancer in the pre-MRI era

Urologists and oncologists gauge prostate cancer aggressiveness by combining DRE findings, serum PSA levels and data derived from systematic biopsy findings.

Men with suspected prostate cancer are categorised into risk groups (see EAU risk classification in EAU guidelines on prostate cancer [2]). This classification is based on the grouping of patients with a similar risk of biochemical recurrence after radical treatment [3]. Tables and nomograms have been developed to predict the likelihood of extraprostatic spread, seminal vesicle invasion and lymph node involvement, and some even state recurrence-free survival rates at 3 and 5 years [4–8].

There is, however, still no consensus of what constitutes a clinically significant prostate cancer (csPCa) [9]. Current argument centres on Gleason category 7 pattern 3 or 4 dominance, ISUP grades 2 and 3, respectively. While the 2014 grading system differentiates Gleason 7 by dominant pattern, all Gleason 7 is classed intermediate risk, albeit with the qualification that emerging clinical data support the distinction between favourable (ISUP grade 2) and unfavourable-risk (ISUP grade 3) patient categories within the intermediate-risk group [2, 10, 11].

### Evaluating csPCa in the post-MRI era

mpMRI can detect and localise cancers with a Gleason score ≥ 7 more easily than lower-grade cancers [12–15], relying on the lower signal intensity of higher-grade cancers on T2-weighted imaging (T2w), more impeded diffusion on diffusion-weighted imaging (DWI), early enhancement on dynamic contrast sequences (DCE), and (previously) higher choline over citrate ratios on spectroscopic imaging [16–18]. mpMRI evaluates lesion volume with reasonable accuracy, at least for aggressive tumours [19]. In one study, correlations between lesion volume estimated on T2-weighted images, ADC maps, and DCE-MR images with pathology were 0.91 and 0.93, respectively [20].

In 2012, the ESUR proposed a standardised reporting tool called ‘PI-RADS’ (Prostate Imaging Reporting and Data System) [1] in an attempt to align mpMRI findings with the risk of having csPCa. In 2015, an updated version (PI-RADS v2) was published in collaboration with the American College of Radiology and the AdMeTech Foundation [21, 22] (Table 1). PI-RADS 2 has been validated in a meta-analysis of 21 studies including over 3857 patients. This demonstrated a pooled sensitivity of 89% and a pooled specificity of 73% [23].

**Table 1:** Comparison of Prostate Imaging and Reporting and Data System versions 1 and 2 (adapted from Barentsz et al [22])

PI-RADS version 1	PI-RADS version 2
A sum score of 3–15 (20 with MRSI) for T2W + DWI + DCE (+ MRSI) is suggested	1–5 point dominant score
	For peripheral zone, DWI is dominant
	For transition zone, T2W is dominant
Equal role for DCE (5-point scale)	Secondary role for DCE (positive or negative)
For DWI: ADC images are mandatory	For DWI: ADC and high *b* value images (*b* value > 1400) are mandatory
27-sector map	39-sector map
MRSI can be included	MRSI is not included
Size is not used for T2W + DWI	Size (> 15 mm) is used for T2W + DWI to separate PI-RADS scores 4 and 5

The EAU/ESTRO/ESUR/SIOG recommends using mpMRI before repeat biopsy, combining a TRUS-directed diagnostic approach *with the addition of the mpMRI and subsequently targeted biopsies* [2, 24]. Neither the European (EAU/ESTRO/SIOG/ESUR) nor the American (NCCN) guidelines endorse wholeheartedly mpMRI in biopsy-naïve men [2, 24].

The NICE guideline CG175 [25] has been updated and is due for publication in April 2019 [https://www.nice.org.uk/guidance/indevelopment/gid-ng10057]; it recommends pre-biopsy mpMRI, putting mpMRI as the primary method to investigate those with suspected prostate cancer based on PSA and/or DRE findings. Revised in November 2018, the French guidelines now also recommend pre-biopsy mpMRI for all, including biopsy-naive [26]. Appendix 1, which includes in addition to references cited in the main text citations to PROTECT trial [27], Belgian National Guidance [28] and recently updated French National Guidance [29].

Eligibility criteria to have an mpMRI (in place of biopsy) should be based on the EAU and/or National current recommendations for biopsy referrals. Following suitable clinical evaluation for acute or chronic reasons *not* to be investigated, the reasons to offer mpMRI would mirror those currently used to offer biopsy.

## Pre-biopsy mpMRI advantages

The problem with any approach dominated by TRUS-guided systematic biopsy (SBx) is that it is organ rather than lesion based, introducing two major limitations: overdiagnosis of clinically insignificant prostate cancers (cisPCa) and under-diagnosis of csPCa.

### The case for excluding men from biopsy based on mpMRI

Avoiding or deferring biopsy (possibly indefinitely) if mpMRI suggests low likelihood of csPCa would reduce the burden to men and to their health systems of initial diagnostic workup and low-grade prostate cancer follow-up. Such an approach may also improve the cost efficiency of the diagnostic workup [30]. Accepting that the results are subject to assumptions around test costs, sensitivity of mpMRI-influenced biopsies, and long-term outcomes of men with PCa, recent analysis of a UK population concluded mpMRI first followed by up to 2 rounds of biopsy is more cost effective than current practice [31]. This strategy requires a high negative predictive value (NPV) of mpMRI in excluding csPCa.

A recent systematic review (9613 men) in conjunction with the EAU-ESTRO-ESUR Prostate Cancer Guidelines panel revealed a median mpMRI NPV of 82% (interquartile range (IQR), 69–92%) for overall cancer exclusion and of 88% (IQR, 86–92%) for csPCa exclusion [32]. The critical issue highlighted in this review is that the reported range of the NPV for mpMRI is extreme and varies according to definitions and risk categorisation used.

A key variable of the NPV is the prevalence of cancer within the population being monitored: when the prevalence doubles from 30 to 60%, the NPV of mpMRI (scores 1–2 taken as ‘negative’) falls from 88 to 67% (for any cancer grade) (Table 2) [32]. NPV therefore is bound to be variable as it depends on whether mpMRI is being used in a low-risk screening setting or in a selected high-risk cohort. Furthermore, the prevalence will alter according to the definition of csPCa (Table 3).

**Table 2:** Negative predictive estimates for pre-biopsy mpMRI as a function of prostate cancer prevalence (adapted from Molovan et al [32])

PCa prevalence	NPV
0.30	0.88 (0.77–0.99)
0.40	0.82 (0.70–0.94)
0.50	0.76 (0.64–0.88)
0.60	0.67 (0.56–0.79)
0.70	0.57 (0.47–0.67)

**Table 3:** Diagnostic accuracy results from mpMRI for different definitions of clinically significant prostate cancer (adapted from PROMIS study [33])

Definition of csPCa	Prevalence (%)	Sensitivity	Specificity	PPV	NPV
Gleason score ≥ 3 + 4 or cancer core length ≥ 4 mm,	57 (53–62)	87 (83–90)	47 (40–53)	69 (64–73)	72 (65–79)
Gleason score ≥ 3 + 4	53 (49–58)	88 (84–91)	45 (39–51)	65 (60–69)	76 (69–82)
Gleason score ≥ 4 + 3 or cancer core length ≥ 6 mm	40 (36–44)	93 (88–96)	41 (36–46)	51 (46–56)	89 (83–94)

Three pivotal multicentre trials on the use of mpMRI in biopsy-naïve men inform this review: PROMIS, PRECISION [33, 34], and the 4M study by van der Leest [35].

The PROMIS trial assessed mpMRI, 12-core SBx and template transperineal biopsy (TTP Bx) in 576 prospectively included biopsy-naïve men [33]. Forty percent of patients had csPCa (defined as Gleason score ≥ 4 + 3 or cancer core length ≥ 6 mm) at TTP (Table 3). Using TTP Bx as a reference test, the NPV for detecting csPCa was 0.89 (95%CI, 0.83–0.94) for mpMRI compared with 0.74 (0.69–0.78) for TRUS SBx (csPCa prevalence,: 40% (95%CI, 36–44%)). mpMRI failed to report 7% (17/230) Gleason 3 + 4 cancers with core lengths between 6 and 12 mm, but no Gleason 4 + 3 or worse cancers. When accepting missing this 7%, mpMRI (used as a triage test) could have avoided 27% of primary biopsies, while detecting 18% more csPCa and ‘missing’ 5% of cisPCa [33]. The definition of csPCa propagated by the START consortium is GS ≥ 3 + 4 [36]. The usage of this definition in the PROMIS study showed an increase in the prevalence of csPCa to 53% (49–58%); the NPV dropped to 76% (69–82%). mpMRI failed to report 12% Gleason 3 + 4 prostate cancers, but still could have avoided 27% of primary biopsies.

Similar results were reported in the more recent multicentre, randomised, noninferiority PRECISION trial, in which 500 biopsy-naïve men were randomised to undergo either mpMRI with or without targeted biopsy, or standard transrectal ultrasonography-guided biopsy [34]. Using mpMRI as a triage test could have avoided 28% of primary biopsies, while detecting 12% more csPCa (defined as Gleason score ≥ 3 + 4) than SBx and ‘missing’ 13% of cisPCa. These results were obtained in 25 centres (academic and non-academic) with mixed experience in both mpMRI and MR-targeted biopsy, and without restrictions on the use of a 1.5-T or a 3.0-T system, endorectal coil, or biopsy technique (visual registration, software-assisted registration or in-bore).

The 4M study included 626 biopsy-naïve patients; all patients underwent systematic biopsy, and those with a positive mpMRI (PI-RADS 3–5, 51%) underwent additional in-bore MRI-TBx. SBx performed in PI-RADS 1–2 cases detected csPCa in only 3% of the patients while detecting cisPCa in 20%, with an 89% reduction in total biopsy cores [35].

In a clinical follow-up study (median follow-up of 41 months) of a mixed population of biopsy-naïve, repeat biopsies, and active surveillance (*n* = 300), who had undergone a negative in-bore MR-guided biopsy for PI-RADS 3–5 lesions, only 1.7% (5/300) had csPCa subsequently diagnosed by any kind of follow-up histology in 82 men (any biopsy or radical prostatectomy), and in 218 without any histology confirmation [37]. In another cohort of 1255 patients with negative mpMRI, the csPCa-free survival rates at 48 months were 95% in originally biopsy-naïve patients and 96% in patients with a prior negative biopsy [38].

### Improving detection of csPCa

Pre-biopsy mpMRI in men with suspected prostate cancer is justified further if it improves the detection of csPCa through targeted biopsies of any suspicious lesion suggested by mpMRI.

Radiologic-pathologic correlations with whole-mounts have shown that mpMRI is highly sensitive for locating aggressive cancers, with 80–86% of Gleason 7 and 93–100% of Gleason ≥ 8 detected [12]. Correlation studies of mpMRI with TBx or radical prostatectomy specimens performed after the introduction of PI-RADS showed that the location of the index lesion was correctly assessed by mpMRI in 95% of patients [39] and that mpMRI missed 10% csPCa on a per-lesion basis [40]. mpMRI results, whether expressed as subjective (Likert) scoring [41–43], PI-RADS v1 [44, 45], or PI-RADS v2 scoring [46], were found significant predictors of the presence of csPCa at biopsy.

A recent systematic review of mostly retrospective studies showed that TBx performed under MR/TRUS fusion detected more csPCa than SBx, with a median detection rate of 33.3% (range, 13.2–50%) versus 23.6% (range, 4.8–52%), respectively. The absolute difference in the detection rates between the two approaches was a median of 6.8% (range, 0.9–41.4%) and always in favour of TBx. The median number of biopsy cores to detect one man with csPCa was 37.1 (IQR, 32.6–82.8) and 9.2 (IQR, 4–37.7) for SBx and TBx, respectively [47]. Another systematic review (focussing on MRI positive men only), including studies that used MR/TRUS fusion, cognitive guidance, or in-bore guidance for TBx, also found that TBx has a higher rate of detection of csPCa than SBx with a sensitivity of 0.91 (95%CI, 0.87–0.94) and 0.76 (95%CI, 0.64–0.84), respectively, in a mixed population of biopsy-naïve men and men with previous negative biopsies [48]. The sensitivity (detection) ratio was 1.10 (95%CI, 1.00–1.22) and significantly in favour of TBx for biopsy-naïve men only, and 1.54 (95%CI, 1.05, 2.26) in men with previous negative biopsies. In a head-to-head comparison in 223 men with elevated PSA and/or abnormal DRE, mpMRI-influenced biopsy outperformed systematic 12-core TRUS biopsy in detecting csPCa on a patient basis (42% vs 35%) and on a lesion basis (74% vs 61%) with a ‘miss rate for significant lesions’ of ~ 18% in the MRI biopsy group versus ~ 26% rate in the TRUS biopsy group [49].

### Targeted versus systematic biopsy versus combined approach for clinically significant prostate cancer detection

mpMRI-targeted biopsies detect about 90% of all csPCa [12, 33, 48]. This, however, also means that about 10% of csPCa are missed if only a targeted approach is adopted. Indeed, histological correlation highlights that Gleason ≥ 7 cancers can be invisible on mpMRI [12]. It therefore may seem prudent, on first thoughts, to supplement targeted biopsies with systematic biopsies to ‘capture’ any csPCa that is missed by mpMRI (usually low grade 4 and organ-confined [50], located in the dorsolateral or apical segments of the peripheral zone [49]).

The combination of SBx and MRI-targeted biopsies (TBx) comes at a cost: over-detection of cisPCa [51, 52]. In a systematic review of 16 studies comparing TBx and SBx in mixed populations of biopsy-naïve men and men with previous negative biopsies, this overdiagnosis was almost halved by omitting SBx [48]. In the PRECISION trial, 13% (95% CI, − 19 to − 7%; *p* < 0.001) fewer men were diagnosed with cisPCa in the MRI-targeted biopsy group (in total 9%) than in the standard biopsy group (in total 22%); again, this diagnosis was more than halved by omitting SBx. Likewise, in a prospective non-randomised trial of 1003 men who underwent both TBx and SBx, adding SBx to TBx identified an additional 103 (22%) prostate cancers, 83% of which were low-grade [53].

Excluding SBx in the MRI-negative men in the 4M would have avoided biopsies in 49% in this study population at a small expense of missing csPCa [35].

The MRI-FIRST multicentre study [54] recruited biopsy-naïve men (*n* = 251) under 75 years with a PSA ≤ 20 ng/ml. All patients had 12-core SBx plus 2 optional cores to hypoechoic lesions by one operator blinded to mpMRI results, and TBx (up to 2 targeted lesions, 3 cores per lesion) by another operator. SBx and TBx detection rates for ISUP grade ≥ 2 tumours were 29.9% and 32.3%, respectively (*p* = 0.38; detection ratio 1.08). ISUP grade ≥ 2 cancers would have been missed in 7.6% (95%CI, 4.6–11.6%) of patients if TBx had not been taken, and in 5.2% (95%CI, 2.8–8.7%) of patients if SBx had not been performed. TBx detected significantly more ISUP grade ≥ 3 tumours than SBx (19.9% vs 15.1%, *p* = 0.0095; detection ratio, 1.32). ISUP grade ≥ 3 cancers would have been missed in 6.0% (95%CI, 3.4–9.7%) of patients if TBx had not been taken, but in only 1.2% (95%CI, 0.2–3.5%) of patients if SBx had not been performed. These data indicate that predominantly ISUP grade 2 prostate cancers were detected by the inclusion of SBx.

Technology that allows mapping exactly the biopsy needle path has suggested that the median number of systematic cores sampling the same region selected for target is 2. If these ‘isometric’ systematic cores are disregarded, systematic biopsy has a modest benefit only: 3% cancer detection instead of benign diagnosis and ~ 1% from cisPCa to csPCa [55].

There are few data comparing TBx to TTP Bx. TTP Bx should be considered in patients at high risk with negative mpMRI and in some patients at low-risk with persistently elevated PSA and a negative MRI.

### Biopsy strategy in the ‘mpMRI first’ era

It is impossible to brush aside that the range of published mpMRI NPV figures is broad and that csPCa prevalence (i.e. pre-MRI probability of csPCa) has a major impact on NPV [9, 32]. Therefore, there is a need to refine the biopsy planning process by incorporating the mpMRI findings within a larger nomogram containing clinical data to determine an individual’s likelihood of having csPCa.

In a recent multivariate logistic regression analysis to predict likelihood of csPCa for biopsy-naive and previously biopsied men, the PI-RADS classification contributed significantly to a newly developed risk model (*p* < 0.001) in combination with the ERSPC-RCs (www.prostatecancer-riskcalculator.com) based on the European Randomised Study of Screening for PCa (ERSPC) [56, 57]. For biopsy-naive men, the risk model reached a higher AUC (0.83), compared with ERSPC-RC3 (0.81), refitted RC3 (0.80), and PI-RADSv1.0 (0.76). The risk model AUC was comparable with that of ERSPC-RC3 + PI-RADSv1.0 (0.84). Likelihood ratio test results similarly showed that the risk-models may perform significantly better compared with (refitted) ERSPCs and PI-RADS alone. Others have confirmed these results [58].

### PI-RADS 3 lesions and risk stratification and biopsy

PI-RADS assessment category 3 is assigned when the probability of prostate cancer is uncertain. The percentage of patients assigned a PI-RADS assessment category of 3 is extremely variable in the different published cohorts [59]. As expected, the biopsy positivity rate is also highly variable in these lesions; the cumulative total of high-grade PCa (GS ≥ 3 + 4) in the PI-RADS category 3 has been reported 21% (range 4–27%) in biopsy-naïve men and 16% (range 10–19%) men with previous negative biopsies [59]. Another paper lists overall cancer detection 16–67% and proportion of Gleason ≥ 7 cancers 0–43% [46]. Based on these data, the authors concluded that the prevalence of PI-RADS 3 index lesions in the diagnostic workup is not negligible, varying between one in five (22%) and one in three (32%) men, depending on patient cohort of the first biopsies or previous negative biopsies. The actual prevalence of csPCa after TBx in PI-RADS category 3 lesions varies between patient groups from one in five (21%) and one in six (16%), depending on previous biopsy status. Although this prevalence is lower in comparison to PI-RADS category 4 and PI-RADS category 5 lesions, still a considerable proportion of men harbour significant disease.

### Biopsy strategy and patient risk

Biopsy decisions should first be based on mpMRI findings, favouring avoiding biopsy in ‘negative’ (any/all lesions PI-RADS 2 or less) studies and targeting PI-RADS 4 or 5 lesions. For some ‘negative’ studies and most PI-RADS 3 lesions, a second assessment incorporating clinical (age, DRE, family history for example) and biochemical (PSA density and velocity) parameters should be applied to see if systematic biopsy alone or in addition to targets to the low-grade PI-RADS lesions are indicated.

The use of PSA density may improve the patient selection for biopsy [32, 35, 56, 57, 60–65]. Two studies using either the PI-RADS V1 scoring [66] or a mix of PI-RADS v1 and v2 systems [37] suggested that PSA density could discriminate among PI-RADS 3 patients, those who need to undergo prostate biopsy from those who can be followed up. In a multicentre study of biopsy-naïve men, PI-RADS category 3 lesions were further categorised into PSA density of < 0.10, 0.10–0.20, and > 0.20: the detection of GS ≥ 3 + 4 PCa was 18%, 31%, 46%, respectively [67].

**Fig. 1:**
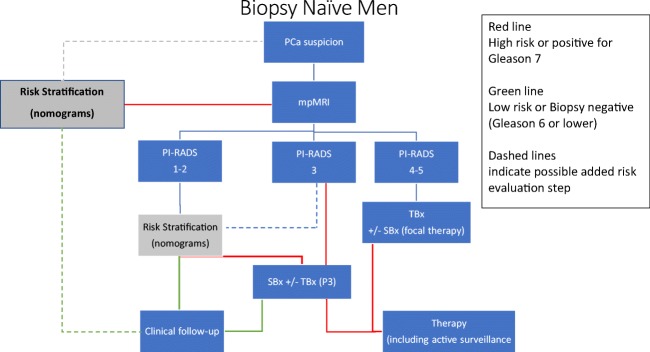
Proposed flowchart for investigating men suspected having prostate cancer, beginning with mpMRI. Using mpMRI as the primary investigation in prostate cancer diagnostic workup following clinical suspicion, men will be stratified into PI-RADS assessment categories 1–2, 3, and 4–5. Capitalising on the high negative predictive value of mpMRI, assessment category 1–2 may indicate clinical follow-up avoiding systematic biopsy, or indicate further risk stratification with developing risk calculators (nomograms). Assessment category 3 may indicate MR-targeted biopsy (TBx) combined with systematic biopsy (SBx) to gain maximal diagnostic yield. Alternatively, risk stratification may sub-differentiate these men into high-risk and low-risk; the low-risk group may defer systematic biopsy. Assessment category 4–5 may indicate MR-targeted biopsy. Systematic biopsy could be performed in direct combination or secondary, depending on biopsy workflow. In assessment category 5, the added value of systematic biopsy would be limited. When prostate cancer has not been identified, additional risk stratification could be performed to indicate or avoid additional systematic and targeted biopsy. Green arrows, low-risk; red arrows, intermediate-/high-risk. Dotted lines indicate research in progress. PCa, prostate cancer; MRI, magnetic resonance; PI-RADS, prostate imaging reporting and data system = suspicion MRI score (1–5); TBx, MRI-targeted biopsy; SBx, transrectal/transperineal ultrasound-guided systematic biopsy; AS, active surveillance

In patients stratified into a priori low-to-intermediate risk (Table 1), the mpMRI NPV is probably sufficiently high to avoid SBx in case of negative mpMRI [34]. Systematic biopsy results would be expected to be less influential on patient management in the setting of a positive mpMRI with a suspicious lesion than in a negative mpMRI. In high-risk patients, however, patients with negative mpMRI will probably still need SBx [34]; even in expert centres, mpMRI may ‘miss’ 10–12% csPCa. The recommended shift towards an MRI-directed and risk-stratified approach to seeking csPCa is captured in the flowchart (Fig. 1). A shift in emphasis away from SBx with additional MRI-TBx to MRI-TBx for high-probability mpMRI examinations is recommended, accepting the modest price of missing csPCa [33, 68–75].

## Inter-reader variability

There are three difficulties with the widespread introduction of pre-biopsy mpMRI: the variable NPV of mpMRI, the variable accuracy of using mpMRI with TRUS to target suspicious lesions regardless of their location within the prostate gland, and inter-reader variability. The results from studies addressing the variability between 2013 and 2017 are summarised in Table 4.

**Table 4:** Inter-reader reproducibility of prostate MRI scoring systems

							Inter-reader agreement			
	Pt no	Histology standard	Analysis level	No readers	Reader experience	Cancer prevalence ^(1)^	Metric used	Likert	PI-RADS V1	PI-RADS V2
Rosenkrantz 2013 [76]	70	RRP	Per region (18 regions)	3	6 years each	PZ, 22.1% (279/1260) TZ, 26.5% (223/840) Both, 13.3% (56/420)	Mean kappa across combinations of readers	PZ, 0.56 [0.51–0.61] TZ, 0.59 [0.56–0.62] Both, 0.45 [0.37–0.57]	PZ, 0.51 [0.41–0.49] TZ, 0.29 [0.24–0.34] Both, 0.51 [0.47–0.55]	–
Rosenkrantz 2013 [77]	55	RRP	Per region (18 regions)	3	R1, 6 years R2, 4 years R3, junior	ND	Mean concordance correlation coefficient across combinations of readers	PZ: R1-R2, 0.63 [0.58–0.67] R1-R3, 0.47 [0.42–0.53] R2-R3, 0.54 [0.49–0.59] TZ: R1-R2, 0.52 [0.44–0.59] R1-R3, 0.40 [0.30–0.48] R2-R3, 0.29 [0.20–0.37] Both: R1-R2, 0.61 [0.57–0.64] R1-R3, 0.47 [0.43–0.51] R2-R3, 0.50 [0.45–0.54]	PZ: R1-R2, 0.68 [0.63–0.72] R1-R3, 0.54 [0.48–0.59] R2-R3, 0.47 [0.42–0.52] TZ: R1-R2, 0.38 [0.29–0.45] R1-R3, 0.28 [0.22–0.34] R2-R3, 0.09 [0.05–0.14] Both: R1-R2, 0.61 [0.57–0.65] R1-R3, 0.48 [0.43–0.52] R2-R3, 0.34 [0.30–0.38]	
Vaché 2014 [78]	215	RRP	Per lesion	3	R1, 11 years R2, 1 year R3, junior	Overall cancer: R1, 58.5% (254/434) R2, 59.6% (226/379) R3, 48.3% (187/387) Gleason ≥ 7: R1, 40.1% (187/387) R2, 43.3% (164/379) R3, 36.4% (141/387)	Kappa for pairs of readers	Overall cancer: R1-R2, 0.52 [0.44–0.60] R1-R3, 0.51 [0.43–0.58] R2-R3, 0.47 [0.38–0.55] Gleason ≥ 7: R1-R2, 0.44 [0.33–0.55] R1-R3, 0.50 [0.37–0.64] R2-R3, 0.37 [0.31–0.50]	Overall cancer: R1-R2, 041 [0.34–0.46] R1-R3, 0.44 [0.37–0.50] R2-R3, 0.38 [0.31–0.44] Gleason ≥ 7: R1-R2, 0.38 [0.28–0.47] R1-R3, 0.39 [0.29–0.49] R2-R3, 0.34 [0.28–0.47]	
Thompson 2014 [79]	165	TBx	Per patient	2	> 1000 MRIs each	61.3% (101/165)	Kappa		0.63 [0.52–0.72]	
Renard-Penna 2015 [80]	50^(2)^	TBx	Per patient	2	> 10 years each	ND for the 50 pts. 58.5% for the cohort of 118 pts	Kappa	0.80 [0.69–0.91]	0.73 [0.61–0.85]	
Muller 2015 [81]	101	TBx	Per lesion	5	6 months–12 years	54.3% (88/162)	Mean kappa across combinations of readers			0.46 ± 0.03
Kasel-Siebert 2016 [82]	82^(3)^	TBx	Per lesion	2	R1, 10 years R2, < 1 year	PZ, 69.2% (27/39) TZ, 12.4% (12/97) Both, 28.7% (39/136)	Kappa		PZ, 0.49 [0.30–0.48] TZ, 0.62 [0.46–0.79] Both, 0.55 [0.41–0.68]	PZ, 0.69 [0.56–0.81] TZ, 0.68 [0.45–0.9] Both, 0.68 [0.56–0.80]
Zhao 2016 [83]	372	TBx	Per patient	2	ND	49.7% (185/372)	Kappa			0.48
Rosenkrantz 2016 [84]	120^(4)^	TBx	Per lesion	6	4–9 years	47.6% (30/63)	Mean kappa across combinations of readers^(5)^ Percent agreement^(5,6)^			PZ/PI-RADS ≥ 3, 0.53 PZ/PI-RADS ≥ 4, 0.59 TZ/PI-RADS ≥ 3,0.39 TZ/PI-RADS ≥ 4, 0.51 Both/PI-RADS ≥ 3, 0.46 Both/PI-RADS ≥ 4, 0.56 PZ/PI-RADS ≥ 3, 81.9% PZ/PI-RADS ≥ 4, 80.1% TZ/PI-RADS ≥ 3, 76.4% TZ/PI-RADS ≥ 4, 75.4% Both/PI-RADS ≥ 3, 79.2% Both/PI-RADS ≥ 4, 77.8%
Polanec 2016 [85]	65	TBx	Per patient	2	> 150 MRIs/year each	50.8% (33/65)	Kappa		0.81	0.71
Tewes 2016 [86]	54	TBx	Per patient	2	2–5 years	57.4% (31/54)	Kappa		All lesions, 0.39 Cancers, 0.14 Benign lesions, 0.50	All lesions, 0.56 Cancers, 0.56 Benign lesions, 0.26
Greer 2017 [87]	35	RRP	Per lesion	5	2 expd, 8–15 years 3 less expd, 2 years	Average of 2.1 lesions per patient. Average of 1.7 true positives per patient (81%)	Average index of specific agreement			Overall: global scoring, 0.58 ± 0.04 PI-RADS ≥ 4, 0.72 ± 0.03 Expd: global scoring, 0.70 ± 0.04 PI-RADS ≥ 4, 0.81 ± 0.04 Less expd: global scoring, 0.53 ± 0.04 PI-RADS ≥ 4, 0.68 ± 0.04

*Pts*, patients; *Ref. Std*, reference standard; *RRP*, retropubic radical prostatectomy; *TBx*, targeted biopsy; *expd*, experienced

^(1)^Prevalence for overall cancer, unless specified otherwise

^(2)^Randomly selected from a cohort of 118 pts. with a single lesion prospectively scored Likert ≥ 3/5; these patients were re-evaluated by two readers

^(3)^Patients with at least one lesion prospectively scored PI-RADS V1 ≥ 3/5

^(4)^Retrospective selection of 120 lesions (one per patient) consisting of 15 lesions with Likert scores 2–5 in PZ and in TZ

^(5)^Average value of two interpretation sessions separated by a training intervention

^(6)^Fraction of all 15 possible pair-wise reader combinations with a concordant reading

The conclusions from the studies are succinctly captured by Hansen et al [88]: (1) mpMRI exams are more often called negative in subspecialist reads (41% vs 20%); (2) second readings of prostate mpMRI by subspecialist uroradiologists significantly improve NPV and PPV; (3) reporter experience may reduce overcalling and avoid over targeting of lesions; and (4) greater education and training of radiologists in prostate mpMRI interpretation are advised. Many European countries are, in collaboration with ESUR and EAU, working to address this training need.

## Conclusion

Current national guidelines in Europe highlight the worth of mpMRI in the management of men with suspected PCa. The case for using mpMRI to help in selecting which men with suspected PCa should have a biopsy—and which need not—and to then select the regions of the prostate to biopsy (and which regions can be ignored) is compelling. The evidence base, including level 1 studies, is overpowering as are the arguments for patient benefit, avoiding either biopsy or overdiagnosis of clinically insignificant cancer.

Patients contemplating a biopsy are becoming aware that imaging by means of mpMRI may permit avoidance of biopsy in some cases and targeting in others. These patients will be understandably anxious to avoid the risks of biopsy, or at least minimise the risks by having fewer biopsy samples. Going away from ‘default’ SBx to premeditated TBx judiciously and selectively complimented by SBx using a two-step risk evaluation offers the best compromise to reduce biopsy rates and reduce overdiagnosis of cisPCa while minimising the chances of missing csPCa. The evidence to expect to avoid SBx altogether even in the era of pre-biopsy mpMRI is weak [54].

Our summary suggestions are presented in Table 5.

**Table 5:** Suggestions for the use of mpMRI as a triage test in those with suspected prostate cancer

1. mpMRI should be the first investigation in the workup of men with suspected prostate cancer (Fig. 1)	
2. PI-RADS assessment categories 1 and 2 have a high predictive value in excluding significant disease, and systematic biopsy may be postponed, especially in men with low-risk of disease following additional risk stratification (see 7 below)	
3. PI-RADS assessment category lesions 4 and 5 should be targeted	
4. PI-RADS assessment category lesion 3 may be targeted and systematic biopsied depending on risk stratification	
5. Targeted biopsy (cognitive, MRI/US fusion, or ‘in-bore’) should be available for biopsy of focal lesions	
6. Systematic biopsies in addition to targeted biopsy should be used judiciously rather than as a default, for example in cases being considered for focal therapy or nerve-sparing surgery	
7. Where clinical risk parameters including age, family history, DRE findings, PSA velocity, and PSA density are of concern, SBx should be considered even in the setting of a negative mpMRI	

## Electronic supplementary material

ESM 1 (DOCX 30.8 kb)

## Abbreviations

ADC: Apparent diffusion coefficient
AUC: Area under the ROC curve
CI: Confidence interval
cisPCa: Clinically insignificant prostate cancer
csPCa: Clinically significant prostate cancer
DCE: Dynamic contrast-enhanced
DRE: Digital rectal examination
DWI: Diffusion-weighted imaging
EAU: European Association of Urology
EPE: Extraprostatic extension
ERSPC: European Randomised Study of Screening for PCa
ESO: European Society of Oncology
ESUR: European Society of Urogenital Radiology
GG: Grade grouping
GS: Gleason score
IQR: Interquartile range
mpMRI: Multiparametric magnetic resonance imaging
MRSI: Magnetic resonance spectroscopic imaging
NPV: Negative predictive value
PI-RADS: Prostate Imaging Reporting and Data System
PI-RADS v1: Prostate Imaging Reporting and Data System version 1
PI-RADS v2: Prostate Imaging Reporting and Data System version 2
PPV: Positive predictive value
PSA: Prostate-specific antigen
PSAd: Prostate-specific antigen density
SBx: Systematic biopsies
T2W: T2 weighted
TBx: MRI-targeted biopsies
TBx: Targeted biopsy
TRUS: Transrectal ultrasound
TTP Bx: Template transperineal biopsy
Trial: PRIAS
Trial: PROTECT
Trial: PRECISION
Trial: PROMIS
