# Supplementary material for: The primacy of multiparametric MRI in men with suspected prostate cancer
Source: Eur Radiol. 2019 Jun 6;29(12):6940–52. doi: 10.1007/s00330-019-06166-z (PMC6828624; doi:10.1007/s00330-019-06166-z)
Supplement: Supplementary file 1 — (DOCX 30.8 kb) [file 330_2019_6166_MOESM1_ESM.docx]

Appendix 1

## National Guidelines on prostate cancer: summary

## **UK** NICE (2014) [25]

The 2014 guidance issued by the UK National Institute for Health & Clinical Excellence (NICE) is in the process of early update following the publication of PROMIS [33] and PROTECT trials [27]. Currently the guidance recommends biparametric (or multi-parametric) MRI before second biopsy. The document also opines on men who have chosen an active surveillance regimen. Table 1.

SEE UPDATE https://www.nice.org.uk/guidance/ng131

**German**

<http://www.leitlinienprogramm-onkologie.de/fileadmin/user_upload/Downloads/Leitlinien/Prostata_5_0/LL_Prostata_Langversion_0_5_0.pdf>

The updated version is expected to make the following recommendations:

Primary diagnosis:

a) Recommendation: “In addition to systematic biopsies areas suspicious for PCa on imaging should be targeted by biopsy."

b) Recommendation: "MRI of the prostate should be performed using a multiparametric examination protocol according to current quality standards."

c) Recommendation: "Areas suspicious for PCa on mpMRI should be targeted by biopsy. An additional systematic biopsy should be performed."

e) Recommendation: "MRI performed according to current quality standards can be used for initial ("primary") diagnosis. However, it is not part of routine diagnostics."

f) Recommendation: "A normal MRI (PI-RADS <3) bears a residual risk of clinically significant prostate cancer. Therefore, a systematic biopsy should be offered as an alternative to PSA-based control examinations."

After negative initial biopsy**:**

g) Recommendation: "After negative systematic biopsy MRI according to current quality standards should be performed in case of ongoing suspicion for PCa."

Active surveillance:

h) Recommendation: "Patients considering Active surveillance should undergo MRI. Suspicious areas on MRI (PI-RADS) should be targeted by biopsy."

i) Recommendation: "Suspicious areas on MRI should undergo MR-guided biopsy."

**Belgian [28]**

The guidelines advocate MRI in diagnosis and also as a follow-up tool in active surveillance. However, the document dates back to 2012-2013. **(**KCE_194A_Prostaat%20kanker_0.pdf). mpMRI is only mentioned in connection with optimizing biopsies. The Belgian guidelines conclude that further economic evaluations on the use of MRI and MRI–guided biopsies for prostate cancer diagnosis will be critical to determine whether this is an efficient approach for risk assessment in all or only selected men presenting with prostate cancer.

**The Netherlands**

<https://richtlijnendatabase.nl/richtlijn/prostaatcarcinoom/diagnostiek/beeldvormend_onderzoek.html>

MRI is recommended for a) diagnostic purposes, b) for MRI guided biopsies if either the patient has a strong clinical suspicious of having prostate cancer MRI should be performed before a biopsy, according to the ESUR consensus based guidelines, or the patient has negative prior biopsies and the continuous clinical suspicious of prostate cancer MRI should be done according to their ESUR consensus guidelines.

## **Swedish**

From December 2012, mpMRI has been recommended in all patients with prior negative biopsies and continuing suspicion of PCa, and in men with a family history OR BRCA2 mutation and a PSA>2mg./l are recommended to have mpMRI.

Since 2017, Swedish Guidelines recommend that mpMRI with targeted Biopsies should be performed before active monitoring. If only targeted Biopsies are performed it should ONLY be within a scientific protocoled study

*(from: Landstingens och regionernas nationella samverkansgrupp inom cancervården Prostatacancer Beskrivning av standardiserat vårdförlopp)*

**Norwegian**

<https://helsedirektoratet.no/retningslinjer/nasjonalt-handlingsprogram-med-retningslinjer-for-diagnostikk-behandling-og-oppfolging-av-prostatakreft>; <http://www.helsebiblioteket.no/fagprosedyrer/ferdige/mr-prostata-med-kontrast>;

The national emphasis is to recommend an upfront MRI before biopsy, this is to facilitate a one stop service and diminish re-biopsy rates.

**French** [26; 29].

The guidelines of the French Association of Urology recommend performing a mpMRI before the first set of biopsy or before repeat biopsy in patients with persisting suspicion of prostate cancer and prior negative biopsy. They also recommend mpMRI before confirmatory biopsy in patients considering active surveillance. In all these cases, they recommend combining systematic and targeted biopsy.

The following Countries do not, at the time of writing, have official guidelines: Italy, Switzerland, Spain. Other international groups aim to standardize and elevate the quality of diagnosis and treatment of prostate cancer, such as the European Society of Oncology, but the worthy recommendations are generic and there is a lack of detail surrounding the imaging aspects.

# 
